# Investigation of pathogenesis of hyperuricemia based on untargeted and targeted metabolomics

**DOI:** 10.1038/s41598-022-18361-y

**Published:** 2022-08-17

**Authors:** Nankun Qin, Ming Qin, Wenjun Shi, Lingbo Kong, Liting Wang, Guang Xu, Yuying Guo, Jiayu Zhang, Qun Ma

**Affiliations:** 1 School of Chinese Pharmacy, Beijing University of Chinese Medicine, Beijing, 102488 China; 2 Affiliated Dongzhimen Hospital, Beijing University of Chinese Medicine, Beijing, 100010 China; 3 School of Pharmacy, Binzhou Medical University, Yantai, 264003 China

**Keywords:** Biomarkers, Molecular medicine, Pathogenesis

## Abstract

Hyperuricemia (HUA) seriously harms human health but the exact etiology and pathogenesis of HUA are not fully understood. Therefore, it is still of great significance to find effective biomarkers and explore the pathogenesis of HUA. Metabolomics reflects the influence of internal and external factors on system metabolism, explains the changes in metabolite levels during the development of diseases, and reveals the molecular mechanism of pathogenesis. Metabolomics is divided into untargeted metabolomics and targeted metabolomics according to different research modes. Each other's advantages can be fully utilized by combining the two so that the results of metabolomics research can be consummated. 20 HUA patients and 20 healthy individuals participated in the experiment, and untargeted metabolomics was employed to find 50 differential metabolites in HUA serum samples. Twelve candidate biomarkers were screened based on literature research and ROC Curve analysis for subsequent verification. Based on the UPLC-TQ-MS analysis platform, the targeted metabolomics detection methods were established and the content of 12 candidate biomarkers was precisely quantified. Compare with the results of untargeted metabolomics, the targeted metabolomics results were considered more reliable.

## Introduction

Hyperuricemia (HUA) is a metabolic disease caused by purine metabolism disorder, excessive uric acid production, or reduced excretion, resulting in increased serum uric acid^1^. The diagnostic criteria of HUA are that under a normal purine diet, two fasting serum uric acid (SUA) levels on different days are ≥420 μmol/L in males and ≥360 μmol/L in females^2^. In recent years, with the rapid development of the economy and the continuous improvement of living standards, the prevalence of HUA is on the rise globally, especially in Asia such as China^3^. A large number of epidemiological and clinical studies have shown that HUA shares the same pathogenesis basis with diabetes and hyperlipidemia—metabolic syndrome^4–6^. HUA is closely related to gout and is an independent risk factor for the occurrence and development of diabetes, cardiovascular disease, hypertension, chronic kidney disease, and other diseases^7^. HUA is the fourth "high" after hypertension, hyperlipidemia, and hyperglycemia.

In recent years, metabolomics has developed rapidly in the field of disease research and has been widely used in the study of the pathological mechanism of various diseases^8,9^, the search for disease biomarkers^10–12^, early diagnosis of disease^13^, drug potential targets to explore^14^, disease treatment and prognosis. According to different research models, metabolomics has been divided into untargeted metabolomics and targeted metabolomics^15^. Untargeted metabolomics is a global analysis of all unknown metabolites in a sample, without the presupposition of specific metabolites and bias, the sufficient and complete metabolite information provided by it is a prerequisite for screening effective biomarkers. Targeted metabolomics studies are designed to validate previous scientific hypotheses or possible biomarkers with high accuracy and to conduct more targeted studies. In the analysis of a given variety of known metabolites, it is possible to find abnormal associations in metabolites under specific physiological states. In recent years, studies on HUA based on metabolomics have been widely reported at home and abroad^16–19^. Qin et al.^20^ divided 20 HUA serum samples and 20 healthy serum samples into seven equal samples, which were pre-treated by different solvent systems, and then analyzed on the ultra-performance liquid chromatography-quadrupole time-of-flight tandem mass spectrometry (UPLC-Q-TOF/MS) analysis platform under the same chromatographic and mass spectrometry conditions. Differences in each group at each analysis stage and final pathway analysis results were subsequently compared. The results showed that there were differences in metabolite extraction ability, differential metabolite quality, and metabolic pathway analysis among all groups. Yang et al.^21^ used UPLC-Q-TOF/MS lipidomics method to analyze the changes in serum metabolites in hyperuricemia rats and identified 13 potential biomarkers, which are mainly involved in the glycerophospholipid metabolism pathway and glycosylphosphatidylinositol anchor protein biosynthesis pathway. It has important guiding significance for clinical diagnosis and screening of hyperuricemia.

Currently, research on endogenous differential metabolites or biomarkers of HUA metabolomics is mostly focused on non-targeted metabolomics studies, which may have false-positive results and are not verified by targeted metabolomics. Untargeted and targeted metabolomics have their advantages and disadvantages. The combination of the two can be used as a powerful tool for the discovery and accurate quantification of differential metabolites, and play an important role in the process of target discovery, making the screened markers more accurate and repeatable, and improving the results of metabolomics research. Not targeted and targeted metabolomic analysis method was used to analyze the different biological samples (such as blood, urine, feces, etc.) to discover metabolite differences and metabolic pathway changes closely related to specific disease phenotypes. Further combine with biological research to explore the function of metabolites and the mechanism of the occurrence and development of diseases, discover relevant targets and perform functional verification. Therefore, now more and more studies combine the two methods^22–24^ to obtain more accurate information and experimental results. Metabolomics can provide a new research direction for searching for markers of HUA and exploring metabolic disorders in the development of HUA.

In general, based on nuclear magnetic resonance (NMR), mass spectrum (MS), liquid chromatography (LC), and gas chromatography (GC) has become a mainstream platform for the identification and quantification of metabolites^13,25–27^. Through sample preparation, instrumental analysis, data pretreatment, multivariate statistical analysis, and subsequent functional and pathway analysis, a complete set of metabolomics studies can be realized, so as to reveal physiological function changes, early diagnosis, screening of diseases, and explore disease mechanisms and discover new drug targets^28,29^. Liquid chromatography-mass spectrometry (LC–MS) is a widely used analytical method in untargeted metabolomics. Using high-throughput analytical techniques, LC–MS data could provide a global metabolic profile^30^. Chen et al.^31^ combined untargeted and targeted metabolomics data-dependent acquisition performed on a quadrupole time-of-flight (Q-TOF) MS system enabling the acquisition of a large number of auto-MS/MS spectra. Subsequently, the targeted ion pairs are selected and measured by triple quadrupole QQQ/MS in MRM mode. Xu et al.^32^ based on the detection of amino acids by ultra-efficient tandem mass spectrometry (LC–MS/MS), the correlation between the content changes of amino acids and chemotherapy sensitivity of patients with advanced breast cancer was investigated. Glycine, l-glutamine, and sarcosine were used to guide the prognosis of patients with breast cancer, providing the basis for the optimization of individualized treatment strategies for advanced breast cancer; Yang^33^ based on LC–MS to targeted metabolomic methods, analyzing the characteristic of the metabolism of patients with gastric cancer and normal serum group, the metabolic disorders of gastric cancer patients included phospholipid, cholesterol, and amino acids, among which dihydro cholesterol could be a potential marker for gastric cancer diagnosis. In a word, LC–MS provides a certain basis for the in-depth understanding of HUA, discovery of drug therapy targets, and exploration of etiology and pathogenesis.

In this paper, 12 candidate biomarkers and seven metabolic pathways were screened by non-targeted metabolomics and verified by targeted metabolomics. The results showed that 12 biomarkers and seven metabolic pathways played an important role in the metabolic activities of HUA. Then based on the ultra-performance liquid chromatography triple quadrupole mass spectrometry (UPLC-TQ-MS) analysis platform, with a simulated serum sample added with mixed standards as the research object, the targeted metabolomics detection methods of serum polar metabolites and lipids metabolites were established respectively, and methodological verifications were conducted respectively. Combined with the results of untargeted and targeted metabolomics, the characteristics and activities of 9 biomarkers with the same content concentration change trend were analyzed. In this way, the reliability of targeted metabolomics was verified. Therefore, this research provided reference value for the biomarker research of HUA metabolomics to a certain extent.

## Results

### Multivariate data analysis

**Figure 1:**
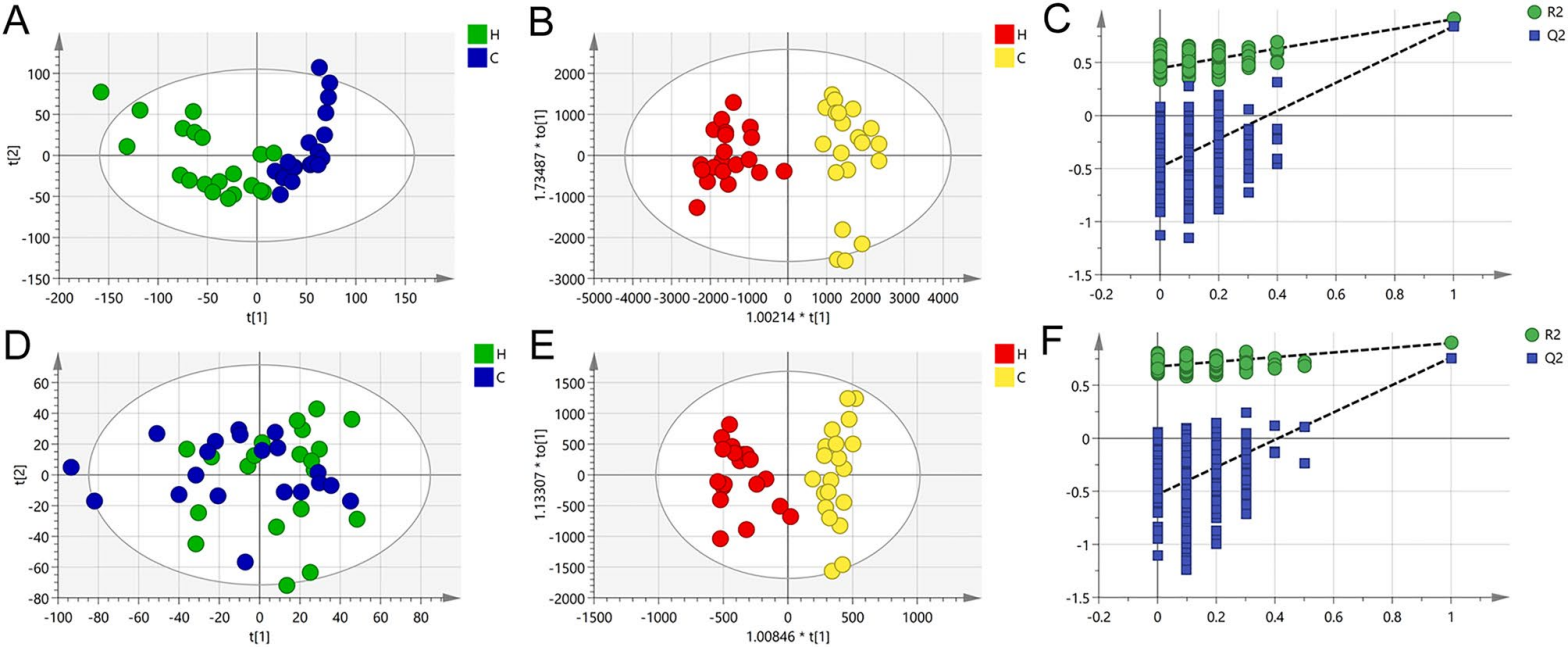
In ESI+ mode and ESI- mode, PCA score, OPLS-DA score, and permutation test of serum samples from the control group and HUA group were performed (*C* control group, *H* HUA group).

Based on LC–MS results of MeOH/MeCN (1:9, v/v) groups’ serum samples, principal component analysis (PCA) was used to study the distribution of metabolites. Figure 1A,D show the groups’ PCA score plots of the control group and HUA group in ESI+ and ESI− mode. According to the PCA score plots, the metabolic patterns of humans behaved differently in different states. It revealed that HUA would cause disturbance in the metabolic pathway in humans.

The orthogonal partial least squares discriminant analysis (OPLS-DA) model is a common fitting model to demonstrate the results of untargeted metabolomics experiments. The OPLS-DA model showed the differences between the HUA group and control group more clearly compared with the results of PCA. The groups’ OPLS-DA score plots of the control group and HUA group in ESI+ and ESI− mode were shown in Fig. 1B,E. The HUA group’s OPLS-DA score plots showed an obvious separation trend from the corresponding control group, which means there was a significant difference in metabolic profiles between the two groups. The values of R^2^Y of the OPLS-DA model were 0.909 (ESI+) and 0.898 (ESI−). The values of Q^2^ of the OPLS-DA model were 0.840 (ESI+) and 0.761 (ESI−). Both of them were higher than 0.755, showing that the established model had a high stability and prediction rate. The permutation test (n = 200) was further used to validate the model, and Fig. 1C,F and are the results of the permutation tests of the MeOH/MeCN (1:9, v/v) group. All R^2^ and Q^2^ values were smaller than the values in the actual model, indicating that there was no overfitting in the OPLS-DA model.

### Metabolites identification and metabolic pathway

There were 461 differential metabolites of MeOH/MeCN (1:9, v/v) group between the control group and HUA group satisfying VIP > 1.0, *P *< 0.05 and log_2_FC > 2.0 or log_2_FC < 0.5. According to the online database, 50 characteristic metabolites of the group inpatient serum metabolic profiles of the group were finally identified, and the results are listed in Supplementary Table S1.

**Figure 2:**
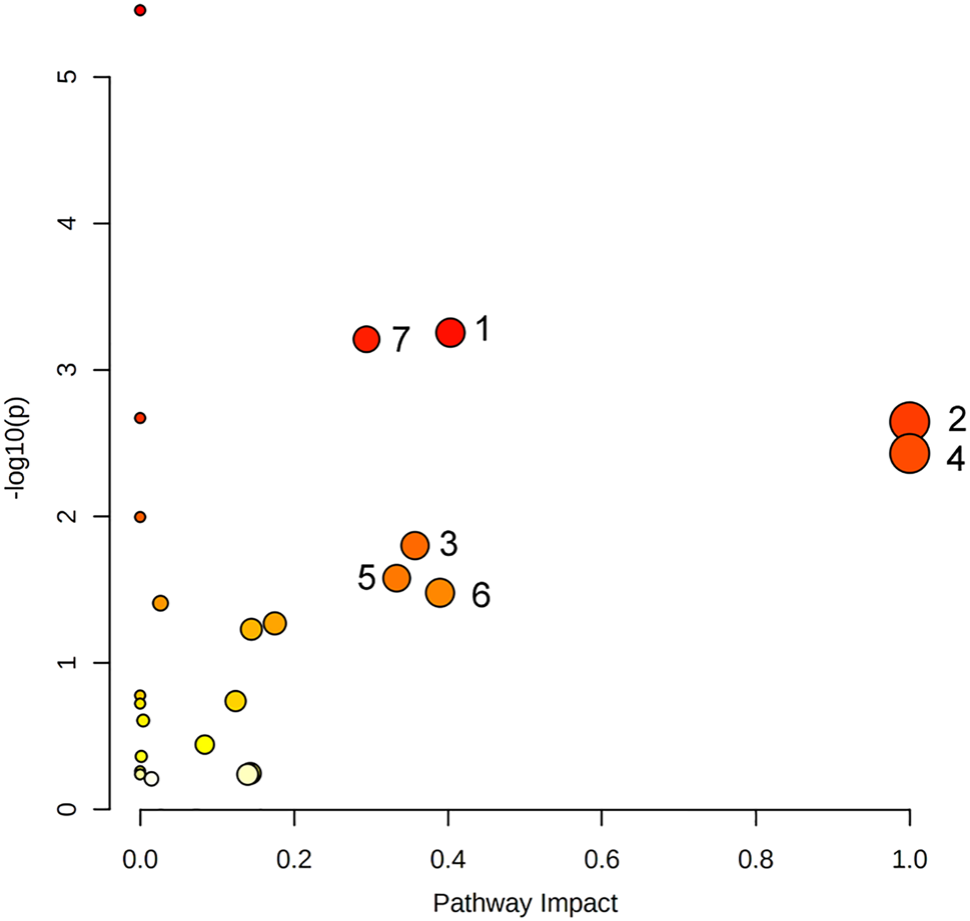
Metabolic pathway analysis of 138 differential metabolites. (1) Glycerophospholipid metabolism; (2) phenylalanine, tyrosine, and tryptophan biosynthesis; (3) phenylalanine metabolism; (4) linoleic acid metabolism; (5) α-linolenic acid metabolism; (6) arachidonic acid metabolism; (7) sphingolipid metabolism.

In order to further explore the overall metabolic changes during the development of HUA, 50 differential metabolites identified in this study were imported into the MetaboAnalyst website for metabolic pathway enrichment and analysis. The significant seven metabolic pathways of serum differential metabolites were shown in Fig. 2, the result implied that multiple metabolic pathways had a certain extent of disturbance effect on HUA. The relevant metabolic pathways are numerically labeled in Fig. 2.

### Screening of candidate biomarkers

**Figure 3:**
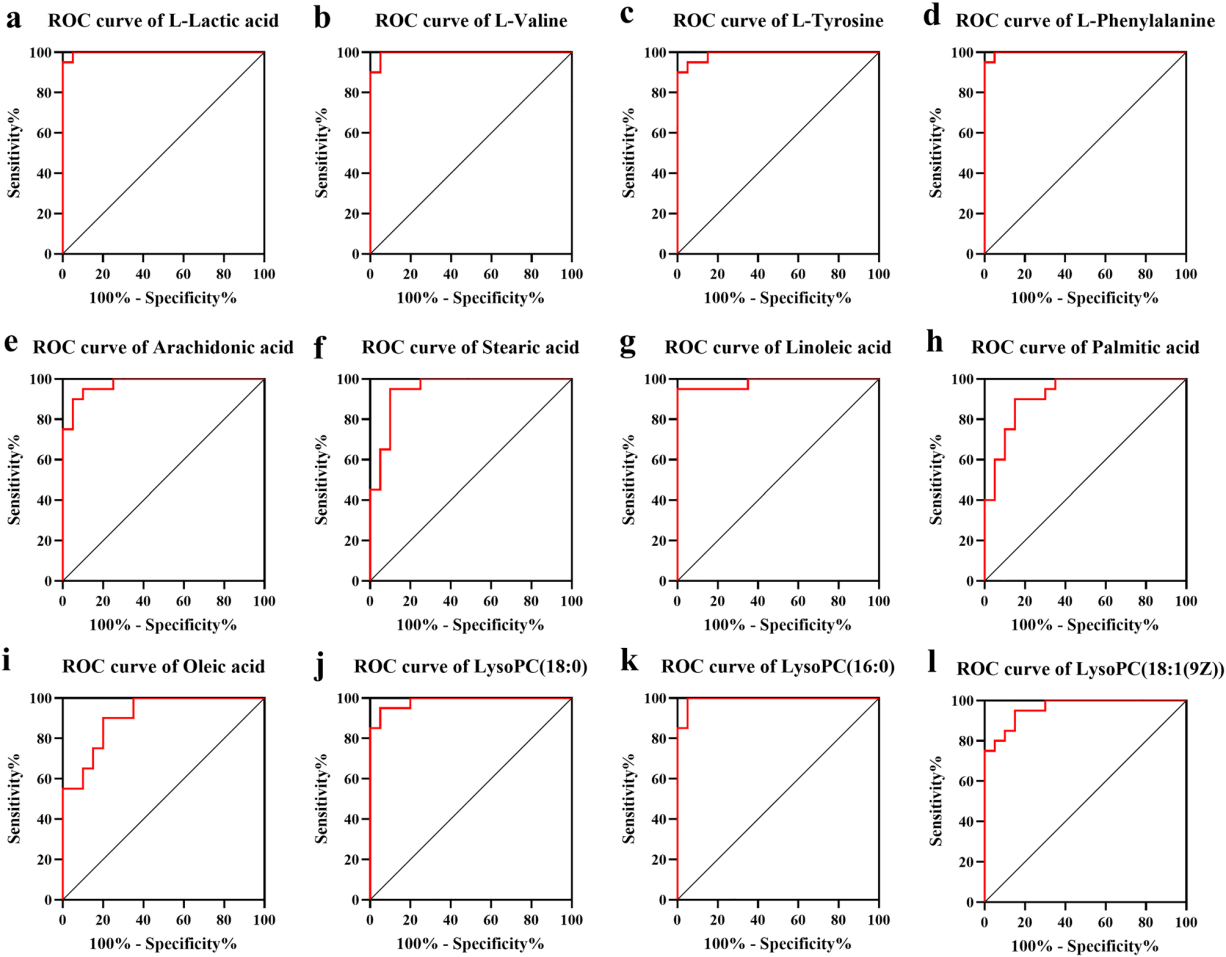
ROC Curve analysis results of candidate biomarkers.

Area under the curve (AUC) values are commonly used to screen candidate biomarkers, 12 differential metabolites with AUC greater than 0.9, which were closely related to HUA and the most relevant metabolic pathways in the literature were identified as candidate biomarkers for further verification, and the specific information is shown in Table 1. The reliability of three biomarkers (l-Valine, l-Lactic acid, and Palmitic acid) with inconsistent content trends was verified according to the results of targeted metabolomics experiments. Among them, AUC results were obtained through Receiver operating characteristic curve (ROC) curve analysis, and ROC Curve analysis results of 12 candidate biomarkers were shown in Fig. 3.

**Table 1:** Target biomarkers for HUA patients.

Substances	Counts	Target biomarkers	Molecular formula	Formula weight (g/mol)	AUC
Organic acids	1	l-Lactic acid	C_3_H_6_O_3_	90.0317	0.9975
Amino acids	3	l-Valine l-Tyrosine l-Phenylalanine	C_5_H_11_NO_2_	117.0790	0.9950
			C_9_H_11_NO_3_	181.0739	0.9900
			C_9_H_11_NO_2_	165.0790	0.9975
Fatty acids	5	Arachidonic acid	C_20_H_32_O_2_	304.2402	0.9750
		Stearic acid	C_18_H_36_O_2_	284.2715	0.9475
		Linoleic acid	C_18_H_32_O_2_	280.2402	0.9825
		Palmitic acid	C_16_H_32_O_2_	256.2402	0.9200
		Oleic acid	C_18_H_34_O_2_	282.2559	0.9100
Lipids	3	LysoPC(18:0)	C_26_H_54_NO_7_P	523.3638	0.9850
		LysoPC(16:0)	C_24_H_50_NO_7_P	495.3325	0.9925
		LysoPC(18:1(9Z))	C_26_H_52_NO_7_P	521.3481	0.9625

### Method validation

The results of specificity, linearity, and lower limit of quantitation (LLOQ), precision and accuracy extraction recovery, and matrix effect, stability are shown in supplementary materials.

### Basic characteristics and biochemistry results

**Figure 4:**
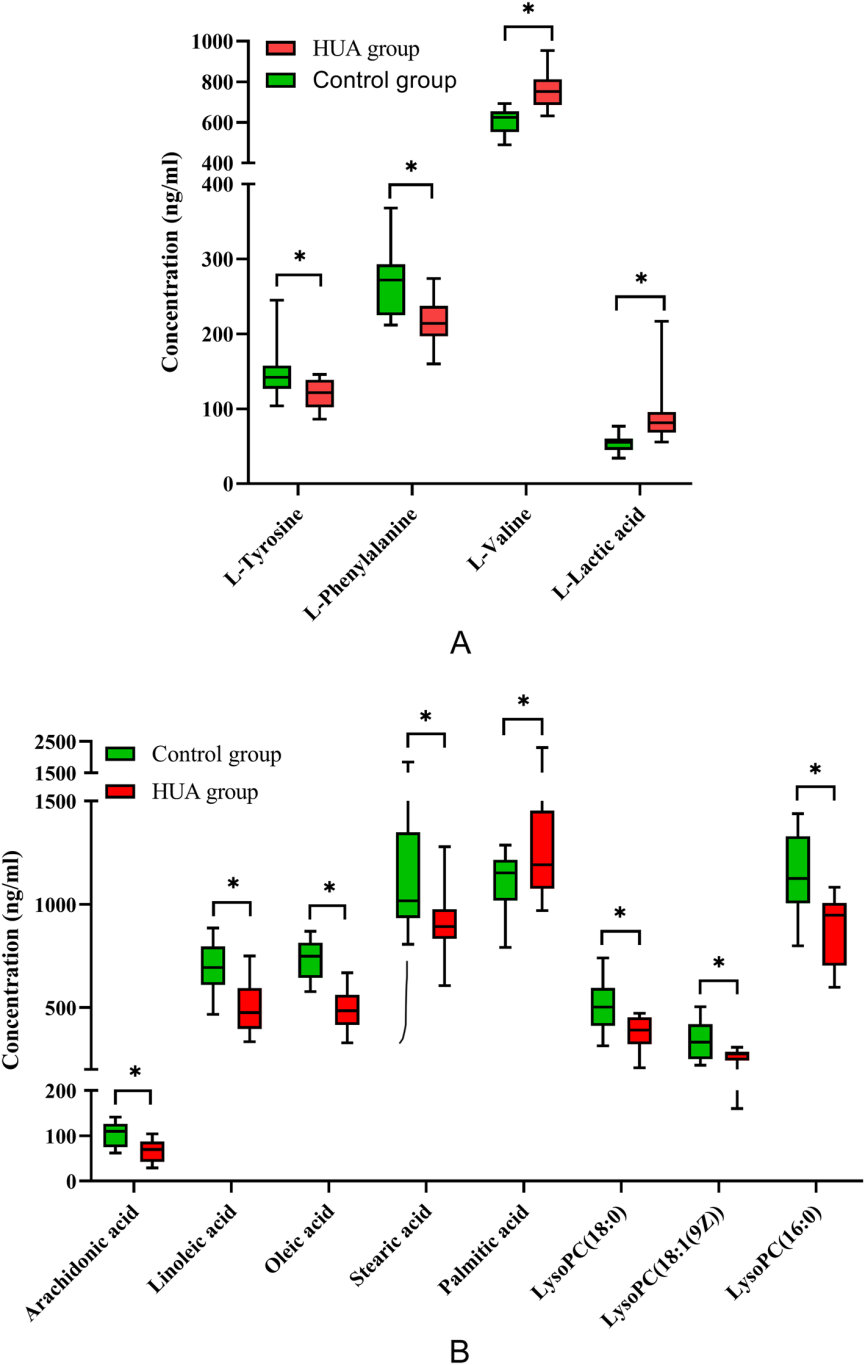
Determination results of serum samples of patients with HUA and normal people (n = 20) [(**A**) polar biomarkers; (**B**) lipid biomarkers] (note: compared with the normal group, *P < 0.01).

HUA patients’ serum samples and normal serum samples’ concentration trends in untargeted and targeted metabolomics and their average target material content determination results as shown in Table 2, and basic characteristics and serum biochemistry results of participants in the control group and HUA group were presented in Fig. 4. The control group included 20 participants, with a mean age of 40.3 ± 11.6 years, 55% were male. The mean age of 20 participants in the HUA group was 41.1 ± 12.6 years, and 55% were male. There was no significant difference (*P *> 0.05) between age and gender. It means that the distribution of age and sex in each group is relatively balanced, which can eliminate the influence of age and sex on the measured indexes to a certain extent. Compared with the control group, l-Tyrosine and l-Phenylalanine of the patients in the HUA group all decreased significantly (*P *< 0.05), and l-Valin and l-Lactic acid of the patients in the HUA group all increased significantly (*P *< 0.05).

**Table 2:** The concentration trends in untargeted and targeted metabolomics and determination results of average content of 12 target biomarkers in serum samples of HUA patients and normal people (n = 20).

Target biomarkers	The average content of target substance (ng/mL)		*P*-value	FDR (false discovery rate)	Trend	
	Control group	HUA group			Untargeted metabolomics	Targeted metabolomics
l-Tyrosine	147.60	120.61	0.015784	0.015757	**↓**	**↓**
l-Phenylalanine	266.80	216.85	0.000148	0.008595	**↓**	**↓**
l-Valine	605.25	760.05	0.000000	0.000000	**↓**	**↑**
l-Lactic acid	54.125	92.06	0.000790	0.011459	**↓**	**↑**
Arachidonic acid	102.35	65.86	0.000046	0.007162	**↓**	**↓**
Linoleic acid	690.35	499.25	0.000009	0.004297	**↓**	**↓**
Oleic acid	735.80	486.30	0.000000	0.000000	**↓**	**↓**
Stearic acid	1157.45	904.90	0.002432	0.014324	**↓**	**↓**
Palmitic acid	1116.10	1333.55	0.017189	0.017189	**↓**	**↑**
LysoPC (18:0)	505.65	379.20	0.000315	0.010027	**↓**	**↓**
LysoPC (18:1(9Z))	337.25	260.50	0.000891	0.012892	**↓**	**↓**
LysoPC(16:0)	1138.60	880.05	0.000038	0.005757	**↓**	**↓**

## Discussion

In this research-based on the previous research method ^20^, MeOH/MeCN (1:9, v/v) was used to extract serum samples of HUA patients. The extraction effect was not only better than that of pure solvent but also more complete non-targeted metabolic information could be obtained. 70 differential metabolites were identified by UPLC-Q-TOF/MS analysis platform. Seven metabolic pathways with the most significant changes in differential metabolites were obtained, and 12 biomarkers were screened. Further targeted metabolomics analysis, based on the UPLC-TQ-MS analysis platform, serum targeted polar metabolite and serum targeted lipid metabolite were established respectively, which verified the experimental results of untargeted metabolomics and made them more scientific and reliable. In addition, metabolic analysis of clinical samples can intuitively reflect human metabolism, and the selected biomarkers and metabolic pathways have more clinical guiding significance, which provides a reliable basis for further understanding of the development mechanism, diagnosis, and treatment of HUA.

Untargeted metabolomics is based on global analysis and is non-biased. In this study, metabolite groups were comprehensively and systematically analyzed, and a large number of metabolite data in serum samples were obtained, from which differential metabolites were found. Among a large number of differential metabolites, biomarkers with high reliability were selected. However, the identification of metabolites is complex. In order to avoid false-positive results, targeted metabolomics analysis was carried out for an established group of metabolites, such as biomarkers for untargeted metabolomics screening, and the corresponding standard materials were used for precise qualitative and quantitative analysis.

50 differential metabolites were screened by untargeted metabolomics, among them 20 were significantly increased in HUA patients, and 30 were significantly decreased in HUA patients, which belonged to amino acids, fatty acids, organic acids, and lipids. Further, explore the overall metabolic changes in the development of HUA, and import 50 differential metabolites into the MetaboAnalyst website for metabolic pathway enrichment and analysis. Based on the MetaboAnalyst platform, multiple metabolic pathways can be matched. The differential metabolites were mainly involving the seven metabolic pathways of glycerophospholipid metabolism, sphingolipid metabolism, arachidonic acid metabolism, linoleic acid metabolism, phenylalanine metabolism, phenylalanine, tyrosine and tryptophan biosynthesis, and alpha-linolenic acid metabolism. The above results are consistent with previous studies^20,34^.

The screening of candidate biomarkers should meet the following three conditions:

- AUC greater than 0.9 (high accuracy);
- It has high participation in the metabolic pathway.
- We refer to the frequency mentioned in previous studies^35–38^.

The AUC was greater than 0.9, which was closely related to HUA in literature research, and the differential metabolites most closely related to metabolic pathways could be identified as candidate biomarkers for further verification. A total of 12 biomarkers met the requirements, these target metabolites included all kinds of metabolites screened by untargeted metabolomics, thus verifying the results of non-targeted metabolomics experiments.

Among the 12 biomarkers, l-Tyrosine, l-Phenylalanine, Arachidonic acid, and Linoleic acid showed the same trend in targeted and untargeted metabolomics experiments, and the reliability of nine biomarkers with the same variation trend was verified. But the contents of l-Valine, l-Lactic acid, and Palmitic acid were different, the difference in the results indicate that the accuracy and reliability of untargeted metabolomics for metabolite identification are not high, the repeatability is poor, the linear range is limited, the metabolite confirmation is complex, and there may be false-positive results. 12 biomarkers were validated for reliability, the AUC value of Oleic acid, l-Valine, and l-Lactic acid was higher than 0.9, indicating high reliability; the AUC value of Palmitic acid is between 0.5 and 0.7, which had low reliability; the AUC value of other substances was between 0.7 and 0.9, which had certain reliability.

Untargeted and targeted metabolomics analysis methods are two main metabolomics analysis methods, both of which have their advantages and disadvantages. Untargeted metabolomics analysis focuses on the "global view" and has advantages in screening differential metabolites on a large scale. In this study, candidate biomarkers cannot be obtained without untargeted metabolomics analysis, so it is necessary to identify these compounds in untargeted metabolomics. But at the same time, it has disadvantages in the accuracy of metabolite identification and quantification. The detection target of targeted metabolomics is a group of known metabolites, and the corresponding standard substances are compared with the substances to be measured in the samples. Therefore, the results have high accuracy and repeatability, which can make up for the defects of non-targeted metabolomics detection. This study has a certain reference value for the confirmation of HUA biomarkers and an in-depth study on the occurrence and development of HUA.

The results of untargeted and targeted metabolomics studies are analyzed in depth. For the nine biomarkers (l-Tyrosine, l-Phenylalanine, Arachidonic acid, Linoleic acid, Oleic acid, Stearic acid, LysoPC (18:0), LysoPC (18:1(9Z)), and LysoPC (16:0), which means that biomarkers screened by untargeted metabolomics have been validated in targeted metabolomics studies, and their results are consistent with trends in clinical studies^39–43^. For the three biomarkers (l-Valine, l-Lactic acid, and Palmitic acid) with inconsistent trends in content concentration, the reasons may be the lower accuracy of untargeted metabolomics analysis, the differences in the time and process of sample collection, or other subjective factors. Concentration multiple refers to the concentration of metabolites in the pathological group/healthy people, and concentration multiple is used as a quantitative index for targeted metabolomics analysis. A review of the literature showed that targeted metabolomics results were more reliable when the concentration changes of untargeted and targeted metabolomics were inconsistent. In untargeted metabolomics studies, l-Valine multiples tended to be down-regulated. However, in targeted metabolomics studies, l-Valine concentration multiples tended to be up-regulated (accurate quantitative analysis results showed statistical significance), and literature^44,45^ showed that the results of targeted metabolomics experiments were more accurate and reliable than those of non-targeted metabolomics experiments. In untargeted metabolomics studies, the concentration multiples of l-Lactic acid tended to be down-regulated. In the follow-up studies of targeted metabolomics, the concentration multiples of l-Lactic acid tended to be up-regulated (accurate quantitative analysis results showed statistical significance), and the results of targeted metabolomics were consistent with those of some clinical studies, indicating its reliability^46,47^. The multiples of Palmitic acid concentrations were down-regulated in untargeted metabolomics studies. In targeted metabolomics studies, concentration multiples tended to be upregulated (accurate quantitative analysis results showed statistical significance). Studies showed that HUA interacted with cardiovascular disease, and palmitic acid was closely related to the progression of cardiovascular disease, suggesting that the upregulation of palmitic acid was reliable^48–51^.

According to the screening results of candidate biomarkers, the metabolic characteristics of each substance were analyzed. l-Lactic acid is the product of the metabolism of hypoxic cells. When tissues and organs are filled with low blood flow or cells are starved of oxygen, anaerobic glycolysis accelerates, exceeding the liver's ability to clear it, leading to increased l-Lactic acid levels. HUA patients have a higher risk of cardiovascular disease^49,52^. HUA and cardiovascular disease interact, and studies have shown that palmitic acid is closely related to the progression of cardiovascular disease. l-Valine repairs tissue regulates blood sugar and provides needed energy. Studies have shown that it significantly affects the activities of ALT and AST in serum^44,46^. In this study, ALT and AST levels were significantly increased in HUA patients, suggesting that up-regulation of l-Valine stimulated ALT and AST production. As an important amino acid, l-Tyrosine is involved in various physiological activities in vivo. The synthesis of dopamine, an important neurotransmitter in the human body, and thyroid hormone require the participation of tyrosine, which can also be converted into fumaric acid and acetyl acetate to participate in metabolic activities such as the TCA cycle of the body and provide the energy for the body^53^. l-Phenylalanine is an essential amino acid that is normally metabolized by Phenylalanine 4-hydroxylase to form l-Tyrosine. l-Phenylalanine is a metabolite upstream of l-Tyrosine, and its content change is closely related to l-Tyrosine. The decrease of l-Phenylalanine content most directly affects the synthesis of l-Tyrosine in the body, leading to the decrease of thyroid hormone level and its metabolic activity. In HUA patients, the levels of l-Tyrosine and l-Phenylalanine were down-regulated, indicating that the synthesis of thyroid hormone was affected, leading to decreased immune levels and metabolic disorders in the body. The decrease of Arachidonic acid content will affect the regulation of lipid levels in the body, suggesting that HUA patients have higher risks of cardiovascular and cerebrovascular diseases, diabetes, skin diseases, atherosclerosis, and other diseases. Metabolites of Arachidonic acid are inflammatory mediators of HUA^54–57^. The down-regulation of Arachidonic acid content also suggests that it may be transformed into various inflammatory mediators and cause inflammatory reactions in the body. The downregulation of Linoleic acid and Oleic acid content in HUA patients suggests high risks of hyperlipidemia, atherosclerosis, autoimmune diseases, and inflammation. Stearic acid is at the intersection of metabolic changes and joint inflammation before gout attack^58^. Down-regulation of Stearic acid content in the serum of HUA patients may indicate that the inflammatory mechanism of HUA is different from that of gout. When Stearic acid content is upregulated in HUA patients, there may be a tendency to develop gout^59^. The downregulation of Oleic acid content suggests that HUA patients have a higher risk of hyperlipidemia and cardiovascular and cerebrovascular diseases. Related to the occurrence of inflammation, may cause reduced immunity, and autoimmune diseases, it is also associated with metabolic disorders of HUA. The down-regulation of Linoleic acid content in the serum of HUA patients suggests higher risks of hypertension, angina pectoris, cardiovascular and cerebrovascular diseases, atherosclerosis, hyperlipidemia, and so on. In addition, Linoleic acid deficiency is related to obesity, and it has been reported that obese people have a higher risk of HUA^60–64^. LPCs (Lyso-phosphatidylcholine) are closely related to diabetes^65^, atherosclerosis^66^, dyslipidemia, and cardiovascular diseases^67^, and are mainly metabolized in the liver. The decrease of LPCs content predicts the increased probability of liver diseases such as cirrhosis^68^, fatty liver^69^, and viral hepatitis^70^ in HUA patients.

These biomarkers screened in this study play an important role in human metabolites, and their metabolic disorders suggest that HUA patients have a higher risk of cardiovascular and cerebrovascular diseases, diabetes, and hyperlipidemia. Therefore, it is essential to detect the blood uric acid in the prevention of these diseases, it also makes a positive contribution to preventing and treating hyperuricemia.

## Methods

### Reagents and instruments

LC–MS grade MeOH, MeCN, and formic acid were purchased from Fisher Scientific (Loughborough, UK). Ultra-pure water was purified by a Milli-Q water system (Millipore, Milford, MA, USA). Bovine serum albumin (BSA) was purchased from Sigma-Aldrich (St. Louis, USA), physiological saline (0.9% sodium chloride solution).

l-Lactic acid (CAS: 79-33-4, purity ≥ 98%), l-Valine (CAS: 72-18-4, purity ≥ 9%), l-Phenylalanine (CAS: 63-91-2, purity ≥ 98%), Arachidonic acid (CAS: 506-32-1, purity ≥ 98%), Stearic acid (CAS: 57-11-4, purity ≥ 98%), Linoleic acid(CAS: 60-33-3, purity ≥ 98%), Palmitic acid (CAS: 57-10-3, purity ≥ 98%), Oleic acid (CAS: 112-80-1, purity ≥ 98%), LysoPC (18:0) (CAS: 19420-57-6, purity ≥ 98%), LysoPC(16:0) (CAS: 17364-16-8, purity ≥ 98%), LysoPC (18:1(9Z)) (CAS: 19420-56-5, purity ≥ 98%), Heptadecanoic acid (IS, CAS: 506-12-7, purity ≥ 98%) and 19:0 LysoPC (IS, CAS: 108273-88-7, purity ≥ 98%) were purchased from Shanghai Yuan-ye Biological Technology Co., Ltd. (Shanghai, China).

Instruments used in this study include a vortex mixer (Haimen Kylin-Bell Lab Instruments Co., Ltd., Jiangsu, China), cryogenic super-centrifuge (Thermo Fisher Scientific, USA), nitrogen evaporator (Beijing Chengmeng Weiye Technology Co., Ltd., Beijing, China), UPLC-Q-TOF/MS (Waters Corp., Milford, MA, USA), UPLC-TQ-MS (Waters Corp., Milford, MA, USA).

### Participants

Participants were randomly collected from the rheumatology clinic and physical examination center of Beijing University of Chinese Medicine Affiliated DongZhiMen Hospital (Beijing, China), HUA patients (n = 20) and healthy volunteers (n = 20) were enrolled in this study. Clinical information related to gender, age, and serum biochemical indicators of participants in the control group and HUA group was collected, as shown in Table 3. Inclusion criteria were: (1) serum uric acid level was ≥ 420μmol/L in males and ≥ 360 μmol/L in females; and (2) aged between 20 and 65 years. Exclusion criteria were: (1) pregnant or lactating women; (2) suffering from the disease of the cardiovascular, kidney, or other diseases that will affect the clinical observations and biological indicators, or having metabolic diseases, tumors, and mental disease; (3) patients with HUA caused using the following drugs: thiazide diuretics, furosemide, pyrazinamide, aspirin, and other drugs. These participants had not teak medicines or supplements before they collected serum samples. All serum samples were stored at −80 °C before analysis.

**Table 3:** Basic characteristics and biochemical indexes of participants in the control group and HUA group (*n* = 20).

Parameter	Control group	HUA group
Age (years)	41.1 ± 12.6	40.3 ± 11.6
Gender (female/male)	55%	55%
Fasting blood glucose (mmol/L)	4.9 ± 0.4	5.8 ± 0.6*
Blood uric acid (µmol/L)	320.4 ± 40.3	481.6 ± 51.0**
Triglyceride (mmol/L)	1.52 ± 0.5	3.3 ± 1.2**
Alanine aminotransferase (U/L)	23.6 ± 10.3	40.7 ± 15.9**
Aspartate aminotransferase (U/L)	20.5 ± 5.7	27.7 ± 12.8*
High density lipoprotein cholesterol (mmol/L)	1.5 ± 0.4	1.2 ± 0.2*
Low-density lipoprotein cholesterin (mmol/L)	2.7 ± 0.6	3.6 ± 0.8**
Creatinine (µmol/L)	79.5 ± 8.7	83.2 ± 14.7*

*P < 0.05, there were significant differences compared with the control group.

**P < 0.01, there were significant differences compared with the control group.

^a^The continuous variable is described as the mean (standard deviation) and the categorical variable as the count (ratio).

### Statement

(1) This study was approved by the Research Ethics Committees of Beijing University of Chinese Medicine Affiliated DongZhiMen Hospital. (2) Informed consent was obtained from all subjects. (3) All Methods Were Conducted According to the Declaration of Helsinki Principles.

### Untargeted metabolomics

#### Sample preparation

If a serum precipitation solvent suitable for HUA metabolomics analysis must be recommended, then MeOH/MeCN (1:9, v/v) can be used for analysis^20^. Frozen serum samples were thawed at 4 °C. Then, 300 µL of the MeOH/MeCN (1:9, v/v) was added to 100-µl serum, vortexed for 5 min, and incubated for 10 min on ice; it was then centrifuged at 12,000 r/min for 10 min at 4 °C. All supernatant was evaporated to dryness. Afterward, the residues were reconstituted in 100 µL of 80% MeOH aqueous, vortexed for 5 min, and incubated for 10 min on ice ^20^; then, they were centrifuged at 12,000 r/min for 10 min at 4 °C. The supernatant was analyzed by UPLC-Q-TOF/MS.

#### UPLC-Q-TOF/MS conditions

The chromatographic separation was achieved on an Acquity UPLC^TM^ System coupled to a Xevo G2 Q-TOF/MS with a Waters UPLC BEH C_18_ column (2.1 × 100 mm I.D., 1.7 µm; Waters Corp., Milford, MA, USA) at a column temperature of 45 °C. The mobile phase was composed of 0.2% formic acid aqueous solution (A) and MeOH (B) with the gradient set as follows: 0–1.0 min, 95–95% B; 1.0–2.0 min, 95–2% B; 2.0–13.0 min, 2–2% B; 13–13.5 min, 2–95% B; 13.5–15 min, 95–95% B. The flow rate was 0.40 mL/min, and the injection volume was 2 μL. The autosampler temperature was conditioned at 4 °C. Electrospray ionization (ESI) in positive ion (ESI+) mode and negative ion (ESI−) mode was applied for high-resolution MS detection. The mass range was set at *m/z* 50–1200 Da. The optimized operating parameters were set as follows: ion spray voltage of 3.0 kV, cone voltage of 25 V, cone gas flow of 50 L/h, source temperature of 120 °C, dry gas (N_2_) flow of 10 mL/min, atomization temperature of 450 °C, and 400 °C for ESI+ and ESI−. MS data were recorded in MSE mode. The accurate mass and composition of the relative target ions were calculated with MassLynx V4.0 software (Waters Corp., Milford, MA/USA).

#### Data processing and multivariate data analysis

Raw data were processed by Progenesis QI software (Nonlinear Dynamics, Newcastle upon Tyne, UK) for peak detection, peak alignment and other operations. The peak area of each sample was extracted as a variable, and all samples were normalized by retention time and m/z. Finally, two-dimensional data matrices were generated. These two-dimensional data matrices were respectively imported into SIMCA-P 14.1 software (Umetrics AB, Umea, Sweden) for pattern recognition, and zero value was removed according to the 80% principle for subsequent statistical analysis. Principal component analysis (PCA) revealed the distribution of metabolites in human serum samples. Orthogonal partial least squares discriminant analysis (OPLS-DA) models were constructed to distinguish sample differences and my differential metabolites in massive data. Model evaluation was generally divided into three types: K-fold cross Validation, a permutation test and CV-ANOVA. In this paper, the commonly used permutation test was used to verify the validity of the OPLS-DA model. The contribution rate of a variable is often described by the variable importance of the projection (VIP) value. The greater the contribution rate is, the larger the VIP value is. The VIP values were generated by the OPLS-DA model. Metabolites with VIP > 1, *P* values of t-test (*P*) < 0.05, and a fold change (log_2_FC) of > 2.0 or log_2_FC < 0.5 were selected as differential metabolites.

#### Metabolites identification and metabolic pathway

The chemical information of differential metabolites was searched through the human metabolome database (HMDB; http://www.hmdb.ca/) and METLIN (http://metlin.Scripps.edu). Input the precise molecular mass, ionization method, and addition ion information of differential metabolites into HMDB and METLIN, in accordance with the rule that the deviation of the *m/z* value does not exceed 0.02. The identification results are proved by combining the exact number of charges and the ionization method that meets the experimental conditions. Compare the primary and secondary mass spectra information of the differential metabolites with the theoretical fragments of the HMDB search results, then infer the structure of the compound and the attribution of the fragments to obtain the HUA differential metabolites.

Moreover, for exploring how the major metabolic pathways related to the differential metabolites were affected, metabolic pathway analysis was performed by MetaboAnalyst 5.0 ^71^ platform (http://www.metaboanalyst.ca). All metabolic pathways found displayed their impact values and *P*-values in the form of bubbles. The metabolic pathways with a pathway impact of > 0.2 and *P* < 0.05 were considered the most significant.

#### Screening of candidate biomarkers

The data of the normal and HUA groups were imported into GraphPad Prism 8.0 software to draw ROC curves, according to the movement of cutoff point/cutoff value, multiple pairs of sensitivity and specificity were obtained, with the sensitivity as the vertical axis and the misdetection rate as the horizontal axis Draw ROC Curve at each point, and then calculate the area under the curve, namely AUC. AUC value indicating the ability of a biomarker group to distinguish between two groups (such as experimental and control groups, disease and healthy groups), is usually between 0.5 and 1.0, and the larger the area is, the better the prediction effect is. When AUC > 0.9, the metabolite is considered to have a very high prediction effect on disease and can be used as a potential biomarker for further study. At the same time, differential metabolites that meet AUC > 0.9 and are closely related to HUA and the most relevant metabolic pathway in literature research can be identified as candidate biomarkers for further verification.

### Targeted metabolomics

#### Preparation of calibration solution and quality control (QC) samples

The stock solutions of l-Lactic acid, l-Valine, and l-Phenylalanine were separately weighed and dissolved in Ultra-pure water to obtain final concentrations of 2.5 mg/mL, respectively. And the stock solutions of l-Tyrosine were weighed and dissolved in Ultra-pure water to obtain final concentrations of 0.5 mg/mL.

Ultra-pure water was used to dilute the stock solution to obtain the series of standard solutions at different concentration levels. The l-Lactic Acid, l-Valine, l-Phenylalanine, and l-Tyrosine standard solution was prepared by diluting 80 µL and 400 µL of stock solution with Ultra-pure water to 4 mL at a concentration of 50 µg/mL.

Simulated serum samples: The 500 mg BSA powder was accurately weighed and placed in a 50 mL volumetric flask, diluted with 10 mg/mL BSA saline solution, and fixed to scale. Diluting the corresponding standard solutions with BSA to prepare plasma calibration solutions at final concentrations of 25–1000 ng/mL for l-Lactic acid, l-Valine, l-Phenylalanine, and l-Tyrosine, respectively. The QC plasma samples containing l-Lactic acid, l-Valine, l-Phenylalanine, and l-Tyrosine (50, 200, and 500ng/mL), were prepared in the same manner.

The stock solutions of Arachidonic acid, Stearic acid, Linoleic acid, Palmitic acid, Oleic acid, LysoPC (18:0), LysoPC (16:0), Heptadecanoic acid (IS), LysoPC (19:0) (IS) and LysoPC (18:1(9Z)) were separately weighed and dissolved in methanol to obtain final concentrations of 0.5 and 0.25 mg/mL, respectively. Methanol was used to dilute the stock solution to obtain the series of standard solutions at different concentration levels. The Linoleic acid, Oleic acid, and LysoPC (18:0) standard solutions were prepared by diluting 200 µL of stock solution with methanol to 2 mL at a concentration of 50 µg/mL.

And the Arachidonic acid and LysoPC (18:1(9Z)) standard solution was prepared by diluting 100 µL and 400 µL of stock solution with methanol to 2 mL at a concentration of 25 and 50 µg/mL. Diluting the corresponding standard solutions with BSA to prepare plasma calibration solutions at final concentrations of 25–750 and 50–2000 ng/mL for Stearic acid, Palmitic acid, LysoPC (16:0), and Arachidonic acid, respectively. Diluting the corresponding standard solutions with BSA to prepare plasma calibration solutions at final concentrations of 50–1500 ng/mL for Linoleic acid, Oleic acid, LysoPC (18:0), and LysoPC (18:1(9Z)). The QC plasma samples contain Stearic acid, Palmitic acid, LysoPC (16:0), Arachidonic acid, Oleic acid, LysoPC (18:0), and LysoPC (18:1(9Z)) (100, 250, 500, 625, 1000 and 1250 ng/mL), were prepared in the same manner.

#### Sample preparation

The 60 μL of MeOH/MeCN (1:9, v/v) was added to a 20 µL serum sample, vortexed for 2 min, and then centrifuged at 13,000 r/min for 10 min at 4 °C. All of the supernatants were evaporated to dryness. Afterward, the residues were redissolved in 200 μL of Ultra-pure water, then, they were centrifuged at 13,000 r/min for 10 min at 4 °C. The supernatant was analyzed by UPLC-TQ/MS.

The 30 μL of methanol and 10 μL of IS solution were added to a 10 µL serum sample, vortexed for 5 min, and then centrifuged at 13,000 r/min for 10 min at 4 °C. All of the supernatants were evaporated to dryness. Afterward, the residues were redissolved in 100 μl of methanol aqueous, then, they were centrifuged at 13,000 r/min for 10 min at 4 °C. The supernatant was analyzed by UPLC-TQ/MS.

#### Analytical conditions

UPLC-TQ/MS condition 1: The chromatographic separation was achieved on UPLC-TQ/MS with a Waters Acquity UPLC CSH C_18_ column (2.1 × 100 mm I.D., 1.7 µm; Waters Corp., Milford, MA, USA) at a column temperature of 40 °C. The mobile phase was composed of 0.1% formic acid aqueous solution (A) and 0.1% formic acid acetonitrile (B) with the isocratic elution set as follows: 95% A (0–6 min). The flow rate was 0.30 mL/min, and the injection volume was 2 μL.

Mass spectrometer (MS) was operated in the positive ion (ESI+) mode by multiple reaction monitoring (MRM) of the transition of l-Lactic acid, l-Valine, l-Tyrosine, and l-Phenylalanine (internal standard, IS). MRM ion pair selection is shown in Table 4. The optimal MS parameters were as follows: capillary voltage 3.48 kV; desolvation gas with the flow rate at 1000 L/h; and temperature of the desolvation set at 498 °C, respectively. The optimized cone voltage and collision energy were 6 V, respectively. Data acquiring and processing were conducted through the MassLynx 4.1 software (Waters Corp., Milford, MA, USA).

**Table 4:** Targeted metabolomics analysis MRM ion pairs for polar metabolites detection.

Target biomarkers	Molecular formula	Parent ion (*m/z*)	Daughter ion (*m/z*)	Cone (V)	Collision (V)
l-Lactic acid	C_3_H_6_O_3_	90.9	90.9	70	8
l-Valine	C_5_H_11_NO_2_	118.0	72.1	10	10
l-Tyrosine	C_9_H_11_NO_3_	181.9	135.9	4	10
l-Phenylalanine	C_9_H_11_NO_2_	165.9	119.9	4	8

UPLC-TQ/MS condition 2: The chromatographic separation was achieved on UPLC-TQ/MS with a Waters Acquity UPLC CSH C_18_ column (2.1 × 100 mm I.D., 1.7 µm; Waters Corp., Milford, MA, USA) at a column temperature of 40 °C. The mobile phase was composed of methanol with the isocratic elution set as follows: 100% methanol (0–5 min). The flow rate was 0.30 mL/min, and the injection volume was 2 μL.

Mass spectrometer (MS) was operated in the positive ion (ESI+) mode by multiple reaction monitoring (MRM) of the transition of Arachidonic acid, Linoleic acid, Oleic acid, Stearic acid, Palmitic acid, LysoPC (18:0), LysoPC(18:1(9Z)), LysoPC (16:0), Heptadecanoic acid (internal standard, IS 1) and LysoPC (19:0) (internal standard, IS 2). MRM ion pair selection is shown in Table 5. The optimal MS parameters were as follows: capillary voltage 2.49 kV; desolvation gas with the flow rate at 1000 L/h; and temperature of the desolvation set at 498 °C, respectively. The optimized cone voltage was 7 V, respectively. Data acquisition and processing were conducted through the MassLynx 4.1 software (Waters Corp., Milford, MA, USA), and the content of each metabolite is calculated by the standard curve method.

**Table 5:** Targeted metabolomics analysis MRM ion pairs for lipid metabolites detection.

Target biomarkers	Molecular formula	Parent ion (*m/z*)	Daughter ion (*m/z*)	Cone (V)	Collision (V)
Arachidonic acid	C_20_H_32_O_2_	303.2	259.2	40	10
Stearic acid	C_18_H_36_O_2_	283.3	265.3	40	10
Linoleic acid	C_18_H_32_O_2_	279.0	261.0	40	10
Palmitic acid	C_16_H_32_O_2_	255.2	237.2	40	10
Oleic acid	C_18_H_34_O_2_	281.2	263.2	40	10
LysoPC (18:0)	C_26_H_54_NO_7_P	568.3	508.3	24	14
LysoPC (16:0)	C_24_H_50_NO_7_P	540.4	480.3	20	16
LysoPC (18:1(9Z))	C_26_H_52_NO_7_P	566.3	506.3	16	16
Heptadecanoic acid (IS1)	C_17_H_34_O_2_	269.0	250.9	40	10
LysoPC (19:0) (IS2)	C_27_H_56_NO_7_P	582.4	522.4	24	16

#### Method validation

The methods of specificity, precision and accuracy, extraction recovery and matrix effect, stability are shown in supplementary materials.

#### Data processing

Data acquiring and processing were conducted through the MassLynx 4.1 software (Waters Corp., Milford, MA, USA).

#### Statistical analysis

Statistical significance was determined using Student’s t-test (FDR adjusted), with the levels of threshold set at *P < 0.05. And the plot of data was performed using Graph-Pad Prism (GraphPad Prism version 8.00 for Windows, GraphPad Software, La Jolla California USA, https://www.graphpad.com).

## Conclusion

In recent years, HUA has become a high prevalence of metabolic diseases in the general population worldwide, but it has not yet attracted enough attention. In this study, 12 candidate biomarkers were screened by untargeted metabolomics and verified by targeted metabolomics. Combined with the results of non-targeted and targeted metabolomics, the selectively, linearity, precision, accuracy, LLOQ, matrix effect, and stability of 12 biomarkers were fitted well. l-tyrosine, l-phenylalanine, Arachidonic acid, Linoleic acid, Oleic acid, Stearic acid, LysoPC (18:0), LysoPC (18:1 (9Z)), and LysoPC (16: 0) all showed downregulation trend, which verified the reliability of the same trend of content and concentration of nine biomarkers in untargeted and targeted metabolomics, and analyzed their metabolic characteristics and activities in vivo. Targeted for the targeted and metabolomics is inconsistent with the content and the concentration change trend of three kinds of biomarkers (l-valine, l-lactic acid, and Palmitic acid), in a targeted metabolomics showed a trend of cut, and present a trend of increase in the targeted metabolomics, combined with some literature suggests that targeted metabolomics results more accurate. Therefore, 12 biomarkers played a decisive role in the metabolism of HUA, not only significantly affecting the metabolic activity of HUA, but to a certain extent for HUA biomarkers of metabolomics research provides the reference value, and discusses the change of HUA inside the occurrence and development mechanism, and the possible It provides the research basis for clinical treatment. HUA is involved in the occurrence and development of various diseases, posing a great threat to human life and health. However, due to the limited number of biomarkers studied, the interpretation of the HUA process and mechanism are not comprehensive enough. Subsequent studies can verify more biomarkers by increasing the amount of clinical samples and the screening range of biomarkers, and revealing the relevant molecular biological mechanism combined with other experimental studies.

## Supplementary Information

Supplementary Information.

## Data Availability

The datasets generated during or analyzed during the current study are not publicly available due to this paper involves the confidentiality of the research project, and the research fund is a confidential project, so it will not be disclosed, but are available from the corresponding author on reasonable request.

## Abbreviations

ALT: Alanine aminotransferase
AST: Aspartate transaminase
AUC: Area under the curve
BSA: Bovine serum albumin
ESI: Electrospray ionization
FDR: False discovery rate
GC: Gas chromatography
HMDB: Human metabolome database
HUA: Hyperuricemia
LC–MS: Liquid chromatography–mass spectrometry
LC–MS/MS: Liquid chromatography tandem–mass spectrometry
LLOQ: Lower limit of quantitation
Log_2_FC: Log_2_fold change
LPC: Lyso-phosphatidylcholine
MRM: Multiple reaction monitoring
OPLS-DA: Orthogonal partial least squares discriminant analysis
PCA: Principal component analysis
QC: Quality control
RE: Relative error
ROC curve: Receiver operating characteristic curve
RSD: Relative standard deviate-on
SUA: Serum uric acid
UPLC-Q-TOF/MS: Ultra-performance liquid chromatography-quadrupole time-of-flight mass spectrometry
UPLC-TQ-MS: Ultra-performance liquid chromatography triple quadrupole mass spectrometry
VIP: Variable importance in projection
