# Supplementary material for: Investigation of pathogenesis of hyperuricemia based on untargeted and targeted metabolomics
Source: Sci Rep. 2022 Aug 17;12:13980. doi: 10.1038/s41598-022-18361-y (PMC9386008; doi:10.1038/s41598-022-18361-y)
Supplement: Supplementary file 1 — Supplementary Information. [file 41598_2022_18361_MOESM1_ESM.docx]

**Methods**

**Method validation.** According to the “Guidance for Industry-Bioanalytical Method Validation” recommended by the US Food and Drug Administration to validate the UPLC-TQ/MS method that we established. Method validation was performed to evaluate the specificity, precision, accuracy, linearity, extraction recovery, matrix effect, lower limit of quantification (LLOQ) and stability.

Specificity. The specificity of the method was assessed by comparing the BSA of blank sample, BSA include L-Lactic acid, L-Valine, L-Tyrosine and L-Phenylalanine Stearic acid, Palmitic acid, LysoPC (16:0), Arachidonic acid Oleic acid, LysoPC (18:0) and LysoPC (18:1(9Z)) spiked with them. This test determines where there is potential interference from endogenous substances with BSA and the BSA of blank sample.

Linearity and LLOQ. The linearity of calibration curves for BSA were evaluated by using 12 appropriate concentrations and by plotting the area ratio of analytes to polar metabolites versus the nominal concentration of analytes (x) by using 1/x^2^ weighted least squares linear regression. The LLOQ was defined as the lowest concentration on the calibration curve.

Precision and accuracy. Intra- and inter-day precision and accuracy were assessed by analyzing QC samples at low, medium, and high concentrations, on three successive days. Concentrations were calculated with calibration curves obtained daily. The percentage of relative standard deviation (RSD, %) represented the precision, and the percentage of relative error (RE, %) represented the accuracy. Criteria for precision and accuracy were both RSD (%) and RE (%) of less than 15% (20% for LLOQ).

Extraction recovery and matrix effect. The extraction recovery and matrix effect were determined at low, medium, and high QC levels were detected by comparing the peak area obtained from BSA and Standard aqueous solution with the average peak areas spiked . For extraction recovery, RSD should be above 60%. On the other hand, an matrix effect value (in %) in the range of 85%~115% was considered as indicative of matrix effect.

Stability. The stability was assessed using the QC samples at low, medium, and high concentrations under two conditions: (1) A freeze-thaw cycle stability test was carried out via repeatedly freezing and thawing QC samples for three cycles before treatment; (2) The short-term stability was assessed by leaving QC samples at room temperature for 6 h before extraction.

**Results**

**Method validation**

Specificity. The specificity of the method was tested with the use of BSA and blank matrix added the reference substance and internal standard sample prepared as described in **Preparation of calibration solution and quality control (QC) samples**. The extraction ion flow diagrams in each material channel are shown in **Supplementary** **Fig.S1 and S2**. The results show that the matrix of BSA has no obvious interference at the peak of the substance to be tested and the internal standard substance, indicating that the method has good specificity.

Linearity and LLOQ. Linear curves of 12 biomarkers are shown in **Supplementary** **Fig.S3**. The retention time, linear range and correlation coefficient of the 12 biomarkers are shown in **Supplementary Table S2.** Coefficients of determination for all target analytes presented good regression value and greater than 0.99. Precision and accuracy values of LLOQ for twelve biomarkers are less than 20%.

Precision and accuracy. The results for intra- and inter-day precision and accuracy for the QC samples at three concentrations are shown in **Supplementary Table** **S3**. The results show that the RSD of precision of intra- and inter-day measurements were less than 15%, and the RE values of accuracy of intra- and inter-day measurements were less than 15%. All values were within the acceptable range, indicated that the established method was precise and accurate.

Extraction recovery and matrix effect. The results of the matrix effect and extraction recovery of bimarkers are summarized in **Supplementary Table S4**. The extraction recoveries of eight polar biomarkers were greater than 68.2%, and the matrix effects ranged from 89.3–107%. The extraction recoveries of four lipid biomarkers were great 63.6%, and the matrix effects ranged from 87.2%-109%. All values were within the acceptable range, indicating that the recovery of biomarker was consistent and reproducible, and there was an insignificant matrix effect on the quantification of biomarkers.

Stability. Results of sample stability (n = 5) are summarized in **Supplementary Table S5**. The results showed that biomarkers was stable under all testing conditions, including three freeze-thaw cycles, left at room temperature for 6h. The RSDs and REs of all samples under the above conditions were both within 15 %.


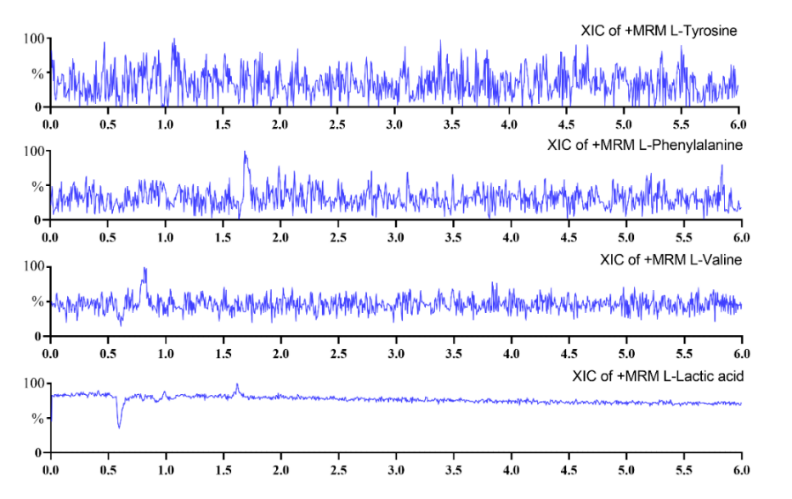


**(A)**


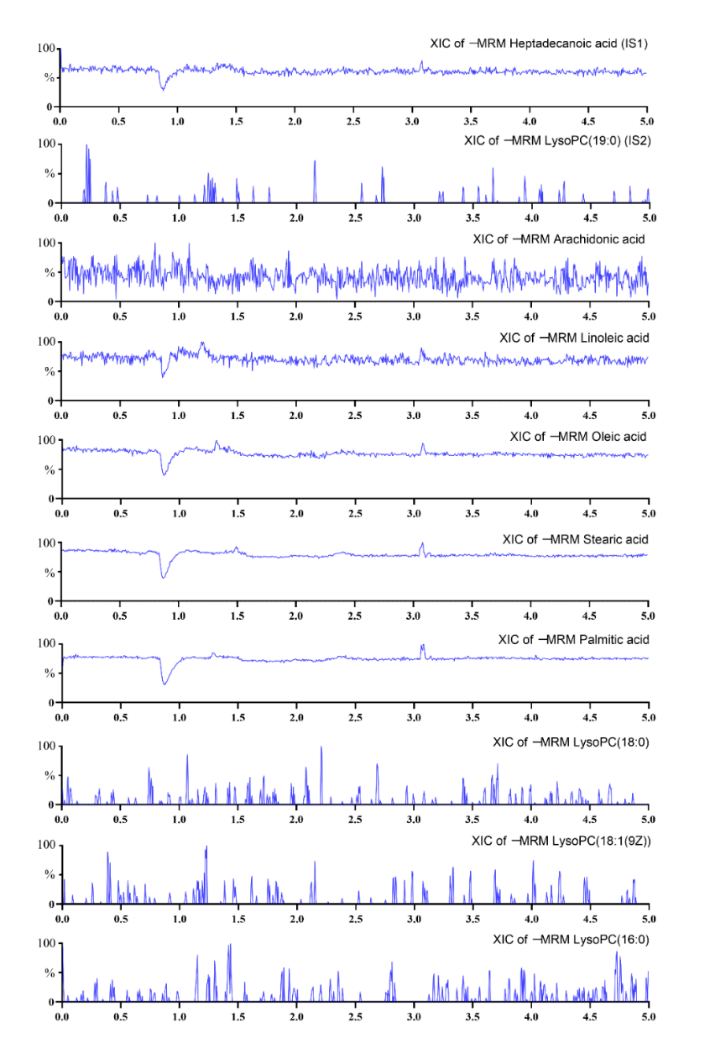


**(B)**

**Supplementary Figure S1.** Extraction Ion Flow Diagram of BSA Blank Samples in Various Substance Channels ((A) Polar biomarkers; (B) Lipid biomarkers)

**
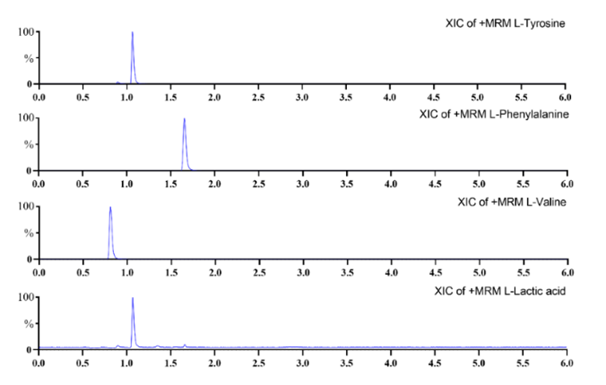
**

**(A)**

**
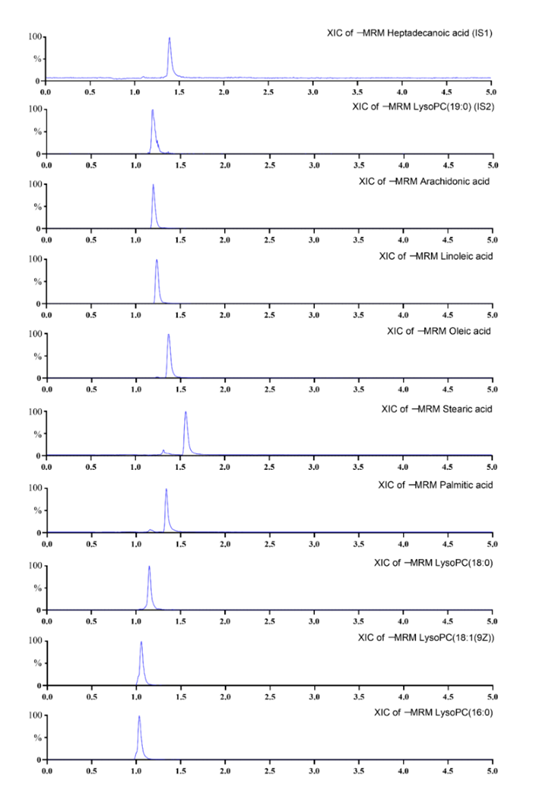
**

**(B)**

**Supplementary Figure S2.** Extraction Ion Flow Diagram of BSA Added Reference Sample and Internal Standard Sample in Various Substance Channels ((A) Polar biomarkers; (B) Lipid biomarkers)

**
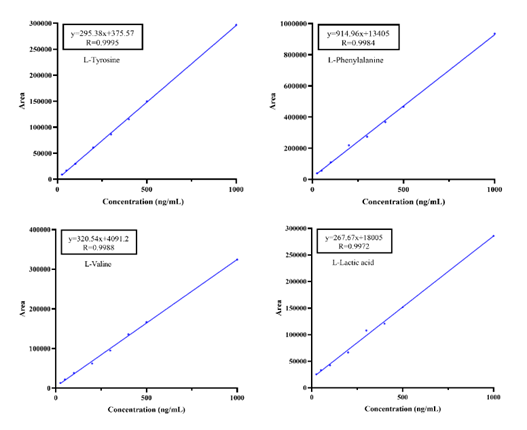
**

**(A)**

**
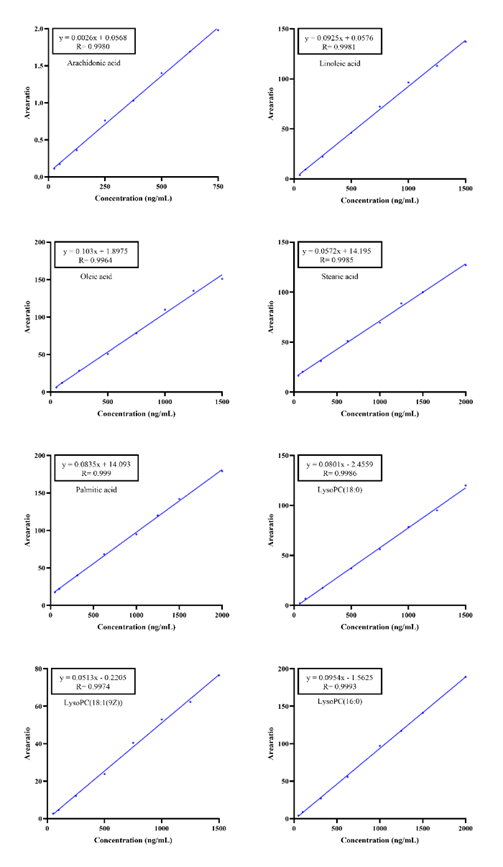
**

**(B)**

**Supplementary Figure S3.** Standard curves of 12 target substances ((A) Polar biomarkers; (B) Lipid biomarkers

| **Supplementary Table S1: The differential metabolites identified of MeOH/MeCN (1:9, v/v) group** | | | | | | | | | | | | |
| --- | --- | --- | --- | --- | --- | --- | --- | --- | --- | --- | --- | --- |
| **No.** | **Metabolites** | **Molecular Formula** | **t_R_ (min)** | **[M+H]^+^/[M-H]^-^** | | **Adducts** | **MS/MS** | **AUC** | **VIP score** | ***P*-value** | **Log_2_FC** | **Trend** |
|  |  |  |  | **Meas. (m/z)** | **Pred. (m/z)** |  |  |  |  |  |  |  |
| 1 | LysoPA(18:1(9Z)/0:0) | C_21_H_41_O_7_P | 12.72 | 435.2520 | 435.2517 | [M-H]^-^ | 435.1658, 171.0127, 152.9978, 96.9636 | 0.9950 | 6.08938 | 1.01E-05 | −1.3187 | ↓ |
| 2 | Phenylalanine | C_9_H_11_NO_2_ | 3.15 | 188.0689 | 188.0682 | [M+Na]^+^ | 103.0543, 91.0544 | 0.9575 | 2.78795 | 8.82E-05 | −2.7355 | ↓ |
| 3 | LysoPC(18:2(9Z,12Z)) | C_26_H_50_NO_7_P | 7.82 | 542.3217 | 542.3217 | [M+Na]^+^ | 502.3284, 258.1130, 184.0735, 125.0001 | 0.9950 | 14.01390 | 1.09E-11 | 1.2681 | ↑ |
| 4 | L-Lactic acid | C_3_H_6_O_3_ | 0.80 | 203.0527 | 203.0526 | [2M+Na]^+^ | 91.0402, 73.0352 | 0.9975 | 3.67120 | 2.11E-11 | −1.0631 | ↓ |
| 5 | L-Valine | C_5_H_11_NO_2_ | 2.64 | 100.0760 | 100.0763 | [M-H_2_O+H]^+^ | 100.0863, 118.0802, 84.9686 | 0.9950 | 5.73410 | 3.68E-11 | −1.2990 | ↓ |
| 6 | Linoleic acid | C_18_H_32_O_2_ | 8.17 | 325.2110 | 325.2114 | [M+2Na-H]^+^ | 191.0914, 177.0083, 125.0116 | 0.9825 | 2.55507 | 5.19E-10 | −1.5545 | ↓ |
| 7 | Oleic acid | C_18_H_34_O_2_ | 8.68 | 623.5202 | 623.5256 | [2M+Hac-H]^-^ | 281.2794, 279.2402 | 0.9100 | 3.98314 | 6.40E-07 | −1.0735 | ↓ |
| 8 | Palmitic acid | C_16_H_32_O_2_ | 8.31 | 301.2109 | 301.2114 | [M+2Na-H]^+^ | 211.0935, 195.1190, 125.0116, 97.1089 | 0.9200 | 2.98999 | 8.71E-07 | −1.2523 | ↓ |
| 9 | LysoPC(6:0) | C_14_H_30_NO_7_P | 8.12 | 373.2120 | 373.2098 | [M+NH_4_]^+^ | 184.0935, 104.1138, 86.1055 | 0.8800 | 2.16634 | 0.0001 | −1.2575 | ↓ |
| 10 | SM(d18:1/24:1(15Z)) | C_47_H_93_N_2_O_6_P | 12.26 | 813.6851 | 813.6844 | [M+H]^+^ | 835.7354, 776.6620, 652.6570, 184.0907 | 0.8125 | 2.08870 | 0.00482 | 1.1119 | ↑ |
| 11 | Arachidic acid | C_20_H_40_O_2_ | 8.33 | 381.2974 | 381.2974 | [M+H+HCOONa]^+^ | 237.1603, 191.0858, 147.0562 | 0.7050 | 4.25671 | 0.00756 | −1.3053 | ↓ |
| 12 | Stearic acid | C_18_H_36_O_2_ | 8.62 | 329.2428 | 329.2427 | [M+2Na-H]^+^ | 195.1162, 96.1068 | 0.9475 | 3.13147 | 0.01255 | −1.6309 | ↓ |
| 13 | LysoPE (0:0/14:1(9Z)) | C_19_H_38_NO_7_P | 5.41 | 424.2453 | 424.2459 | [M+H]^+^ | 167.0692, 77.0114 | 0.9950 | 2.24154 | 4.38E-13 | −1.0534 | ↓ |
| 14 | PGP(18:0/18:1(9Z)) | C_42_H_82_O_13_P_2_ | 5.40 | 821.5126 | 821.5104 | [M+H-2H_2_O]^+^ | 857.5781, 475.2819, 421.2448 | 0.9925 | 1.97061 | 4.91E-13 | −1.0213 | ↓ |
| 15 | TG (16:0/16:0/16:0) | C_51_H_98_O_6_ | 11.53 | 807.7442 | 807.7436 | [M+H]^+^ | 807.7362, 667.5866, 551.5592, 93.0760 | 0.9950 | 1.48619 | 4.57E-12 | −1.2997 | ↓ |
| 16 | PC (24:0/15:0) | C_48_H_96_NO_8_P | 13.77 | 282.9051 | 282.9031 | [M+3H]^+^ | 746.8551, 186.4712 | 0.9950 | 1.74056 | 9.48E-12 | −1.1423 | ↓ |
| 17 | LysoPC (18:0) | C_26_H_54_NO_7_P | 8.18 | 506.3595 | 506.3611 | [M-H_2_O+H]^+^ | 546.4089, 487.3327, 341.3416, 146.9992 | 0.9850 | 1.88144 | 1.14E-11 | −1.1071 | ↓ |
| 18 | LysoPC(18:1(9Z)) | C_26_H_52_NO_7_P | 7.98 | 544.3370 | 544.3374 | [M+Na]^+^ | 544.3931, 485.3164, 339.3266, 184.0935 | 0.9625 | 1.51112 | 5.87E-11 | −1.1542 | ↓ |
| 19 | L-Tyrosine | C_9_H_11_NO_3_ | 1.30 | 182.0813 | 182.0812 | [M+H]^+^ | 95.0598, 108.0581, 165.0686 | 0.9900 | 1.58783 | 1.46E-10 | −1.0210 | ↓ |
| 20 | Arachidonic acid | C_20_H_32_O_2_ | 8.45 | 269.2270 | 269.2275 | [M+H-2H_2_O]^+^ | 287.2590, 245.2448, 205.0569, 191.0858 | 0.9750 | 1.58306 | 1.02E-09 | −1.4292 | ↓ |
| 21 | LysoPC (16:0) | C_24_H_50_NO_7_P | 7.85 | 518.3220 | 518.3217 | [M+Na]^+^ | 459.2830, 313.2975, 146.9942, 104.1164 | 0.9925 | 1.62512 | 6.18E-08 | −1.1920 | ↓ |
| 22 | LysoPC (0:0/16:0) | C_24_H_50_NO_7_P | 7.93 | 478.3300 | 478.3298 | [M+H-H_2_O]^+^ | 496.3735, 478.3660, 184.0879, 104.1143 | 0.9450 | 2.01797 | 7.64E-08 | −1.0190 | ↓ |
| 23 | SM(d18:0/23:0) | C_46_H_95_N_2_O_6_P | 8.58 | 421.3316 | 421.3316 | [M+H+K]^+^ | 620.5383, 600.4996, 337.2687, 184.0851 | 0.9325 | 1.46801 | 4.98E-07 | −1.3635 | ↓ |
| 24 | S1P | C_18_H_40_NO_5_P | 5.90 | 426.2328 | 426.2356 | [M+2Na-H]^+^ | 123.2068, 98.9965, 71.0035 | 0.8875 | 1.59710 | 1.70E-06 | −1.1455 | ↓ |
| 25 | DG(20:0/24:0/0:0) | C_47_H_92_O_5_ | 9.84 | 369.3515 | 369.3545 | [M+2H]^+^ | 447.3812, 391.3652 | 0.9725 | 1.49902 | 1.98E-06 | −1.3318 | ↓ |
| 26 | SM(d17:1/24:1(15Z)) | C_46_H_92_N_2_O_6_P | 11.73 | 799.6695 | 799.6688 | [M+H]^+^ | 821.7209, 762.6400, 638.6345 | 0.9475 | 2.16403 | 1.91E-05 | 1.9118 | ↑ |
| 27 | SM(d18:1/24:0) | C_47_H_96_N_2_O_6_P | 13.63 | 837.6841 | 837.6820 | [M+Na]^+^ | 837.7589, 778.6698, 654.6703 | 0.9275 | 1.95690 | 0.00017 | 2.5204 | ↑ |
| 28 | DG(15:0/24:0/0:0) | C_42_H_82_O_5_ | 8.62 | 334.3166 | 334.3154 | [M+2H]^+^ | 649.5993, 567.4105, 351.3705 | 0.8775 | 1.44697 | 9.75E-12 | −1.1409 | ↓ |
| 29 | LysoPC (15:0) | C_23_H_48_NO_7_P | 8.10 | 464.3165 | 464.3141 | [M+H-H_2_O]^+^ | 258.1264, 184.0879, 104.1134 | 0.9325 | 1.29575 | 0.00019 | −1.7005 | ↓ |
| 30 | Cer(d18:1/24:1(15Z)) | C_42_H_81_NO_3_ | 12.72 | 670.6115 | 670.6109 | [M+Na]^+^ | 282.9311, 264.1986 | 0.8775 | 1.34863 | 0.00082 | 1.3369 | ↑ |
| 31 | PC(14:0/24:1(15Z)) | C_46_H_90_NO_8_P | 11.67 | 816.6481 | 816.6477 | [M+H]^+^ | 628.9125, 413.2895 | 0.8400 | 1.94963 | 0.00314 | 1.5763 | ↑ |
| 32 | Leukotriene A4 | C_20_H_30_O_3_ | 7.51 | 319.2284 | 319.2268 | [M+H]^+^ | 283.0314, 227.9678, 199.0415, 133.0946 | 0.7675 | 1.33102 | 0.00363 | −1.7816 | ↓ |
| 33 | MG(0:0/15:0/0:0) | C_18_H_36_O_4_ | 7.58 | 355.2246 | 355.2245 | [M+K]^+^ | 285.2794, 243.1766, 227.2774 | 0.8725 | 2.06305 | 0.00375 | −3.0977 | ↓ |
| 34 | GlcCer(d18:1/12:0) | C_36_H_69_NO_8_ | 8.76 | 688.5010 | 688.5005 | [M+FA-H]^-^ | 642.5350, 480.4621, 161.0653 | 0.8450 | 1.41797 | 0.01312 | 2.8237 | ↑ |
| 35 | Cer(d18:1/18:0) | C_36_H_71_NO_3_ | 8.50 | 564.5344 | 564.5361 | [M-H]^-^ | 564.3699, 296.2811, 280.2472 | 0.8625 | 2.16102 | 0.00169 | −2.1523 | ↓ |
| 36 | PE(14:0/20:1(11Z)) | C_39_H_76_NO_8_P | 9.84 | 738.5090 | 738.5055 | [M+Na-2H]^-^ | 506.9054, 488.3139, 309.2119, 227.2113 | 0.7525 | 6.70900 | 0.00636 | 1.3623 | ↑ |
| 37 | PC(14:0/16:1(9Z)) | C_38_H_74_NO_8_P | 10.08 | 702.5095 | 702.5079 | [M-H]^-^ | 255.2529, 183.0239 | 0.7350 | 6.14134 | 0.00814 | 1.0341 | ↑ |
| 38 | PE(18:0/18:1(9Z)) | C_41_H_80_NO_8_P | 10.37 | 744.5568 | 744.5549 | [M-H]^-^ | 744.6121, 480.3505, 281.2697 | 0.7175 | 4.38119 | 0.00921 | 1.3239 | ↑ |
| 39 | LysoPA (18:0e/0:0) | C_21_H_45_O_6_P | 7.09 | 423.2879 | 423.2881 | [M-H]^-^ | 269.2313, 96.9657 | 0.7025 | 2.05829 | 0.02091 | 1.1425 | ↑ |
| 40 | LysoPI (18:0/0:0) | C_27_H_53_O_12_P | 7.51 | 599.3213 | 599.3202 | [M-H]^-^ | 437.3986, 316.0688, 283.2893, 153.0057 | 0.6375 | 2.04075 | 0.04179 | 1.0048 | ↑ |
| 41 | SM(d18:1/20:0) | C_43_H_87_N_2_O_6_P | 11.09 | 803.6300 | 803.6284 | [M+Na-2H]^-^ | 656.6307, 434.3095 | 0.8425 | 2.25154 | 0.00339 | 1.1331 | ↑ |
| 42 | LysoPC(22:1(13Z)/0:0) | C_30_H_60_NO_7_P | 8.61 | 560.4063 | 560.4080 | [M+H-H_2_O]^+^ | 481.3681, 184.0907, 104.1164 | 0.8075 | 1.08008 | 0.00097 | 2.9992 | ↑ |
| 43 | PC(P-18:1(9Z)/14:0) | C_40_H_78_NO_7_P | 10.89 | 714.5456 | 714.5443 | [M-H]^-^ | 750.6146, 452.2615, 434.3010 | 1.0000 | 1.38696 | 0.00108 | 2.0902 | ↑ |
| 44 | LysoPC(0:0/18:0) | C_26_H_54_NO_7_P | 8.12 | 1091.7317 | 1091.7258 | [2M+FA-H]^-^ | 463.2351, 419.3439, 283.2790, 168.0531 | 0.9850 | 1.14061 | 0.04617 | −1.1783 | ↓ |
| 45 | SM(d17:1/24:0) | C_46_H_94_N_2_O_6_P | 12.84 | 823.6680 | 823.6663 | [M+Na]^+^ | 764.6693, 640.6365, 184.0879 | 0.9200 | 1.13664 | 0.00127 | 3.371 | ↑ |
| 46 | SM(d18:1/18:0) | C_41_H_84_N_2_O_6_P | 10.29 | 843.5865 | 843.5845 | [M+TFA-H]^-^ | 729.6061, 282.3379, 122.9915 | 0.9125 | 1.68419 | 0.00128 | 1.8463 | ↑ |
| 47 | SM(d18:0/16:0) | C_40_H_74_NO_10_P | 9.90 | 749.5823 | 749.5814 | [M+FA-H]^-^ | 644.6828, 520.6297 | 0.8775 | 1.38543 | 0.0016 | 1.3575 | ↑ |
| 48 | SM(d18:1/22:0) | C_45_H_91_N_2_O_6_P | 12.86 | 845.6787 | 845.6753 | [M+Hac-H]^-^ | 588.6370, 572.5961, 372.2287, 295.3783 | 0.9250 | 1.06529 | 0.00251 | 3.3661 | ↑ |
| 49 | PE(20:0/14:1(9Z)) | C_39_H_76_NO_8_P | 10.39 | 716.5253 | 716.5236 | [M-H]^-^ | 508.3945, 311.2539, 225.0036 | 0.8225 | 1.43738 | 0.00479 | −4.1959 | ↓ |
| 50 | PC(15:0/16:1(9Z)) | C_39_H_76_NO_8_P | 10.44 | 716.5246 | 716.5236 | [M-H]^-^ | 521.4692, 373.2239, 249.1942 | 0.7513 | 1.30086 | 0.01372 | 1.6536 | ↑ |

**Supplementary Table S2**. Retention time, linear range and correlation coefficient of 12 biomarkers

| **Biomarkers** | **Retention time (min)** | **Linear range (ng/mL)** | **Correlation coefficient, R** |
| --- | --- | --- | --- |
| L-Tyrosine | 1.06 | 25-1000 | 0.9995 |
| L-Phenylalanine | 1.66 | 25-1000 | 0.9984 |
| L-Valine | 0.81 | 25-1000 | 0.9988 |
| L-Lactic acid | 1.07 | 25-1000 | 0.9972 |
| Arachidonic acid | 1.16 | 25-750 | 0.9980 |
| Linoleic acid | 1.21 | 50-1500 | 0.9981 |
| Oleic acid | 1.32 | 50-1500 | 0.9964 |
| Stearic acid | 1.49 | 50-2000 | 0.9985 |
| Palmitic acid | 1.26 | 50-2000 | 0.9990 |
| LysoPC(18:0) | 1.12 | 50-1500 | 0.9986 |
| LysoPC(18:1(9Z)) | 1.06 | 50-1500 | 0.9974 |
| LysoPC(16:0) | 1.02 | 50-2000 | 0.9993 |

**Supplementary Table S3.** The precision and accuracy results of low, medium and high concentration QC of 12 target substances

| **Substances** | **Theoretical concentration (ng/mL)** | **Within-day (*n* = 5)** | | **Inter-day (*n* = 15)** | |
| --- | --- | --- | --- | --- | --- |
|  |  | **Precision (CV%)** | **Accuracy**  **(RE%)** | **Precision (CV%)** | **Accuracy**  **(RE%)** |
| L-Tyrosine | 50 | 1.45 | 8.29 | 2.14 | 7.43 |
|  | 200 | 3.44 | 3.59 | 5.52 | 4.68 |
|  | 500 | 1.56 | 1.50 | 1.35 | 2.45 |
| L-Phenylalanine | 50 | 8.57 | 6.26 | 8.79 | 6.55 |
|  | 200 | 1.75 | 13.10 | 3.00 | 12.60 |
|  | 500 | 4.04 | 8.63 | 3.17 | 6.15 |
| L-Valine | 50 | 3.40 | 10.60 | 3.82 | 8.84 |
|  | 200 | 1.77 | 13.70 | 2.34 | 12.90 |
|  | 500 | 1.40 | 1.47 | 2.39 | 3.98 |
| L-Lactic acid | 50 | 10.80 | 10.70 | 6.76 | 9.27 |
|  | 200 | 5.56 | 10.50 | 5.93 | 9.64 |
|  | 500 | 1.30 | 3.74 | 2.17 | 2.63 |
| Arachidonic acid | 50 | 4.65 | 8.71 | 2.82 | 9.31 |
|  | 250 | 3.45 | 2.59 | 4.23 | 4.97 |
|  | 500 | 4.89 | 4.65 | 2.86 | 6.53 |
| Linoleic acid | 100 | 3.51 | 11.20 | 5.99 | 10.40 |
|  | 500 | 3.69 | 3.25 | 4.22 | 6.40 |
|  | 1000 | 3.72 | 7.10 | 3.33 | 4.35 |
| Oleic acid | 100 | 6.54 | 5.10 | 5.76 | 7.84 |
|  | 500 | 4.58 | 5.41 | 3.49 | 8.36 |
|  | 1000 | 9.68 | 7.45 | 6.26 | 6.55 |
| Stearic acid | 100 | 8.75 | 8.75 | 5.42 | 7.81 |
|  | 625 | 4.12 | 3.79 | 3.68 | 4.26 |
|  | 1250 | 8.48 | 5.92 | 7.81 | 6.55 |
| Palmitic acid | 100 | 6.12 | 8.20 | 8.15 | 9.05 |
|  | 625 | 6.42 | 5.65 | 5.72 | 6.38 |
|  | 1250 | 9.23 | 8.67 | 7.51 | 6.83 |
| LysoPC(18:0) | 100 | 2.10 | 11.00 | 2.26 | 10.80 |
|  | 500 | 2.95 | 6.10 | 3.84 | 5.84 |
|  | 1000 | 3.12 | 6.46 | 2.70 | 4.40 |
| LysoPC(18:1(9Z)) | 100 | 5.10 | 11.40 | 4.16 | 10.60 |
|  | 500 | 3.96 | 3.67 | 5.57 | 6.00 |
|  | 1000 | 4.92 | 5.29 | 3.63 | 5.04 |
| LysoPC(16:0) | 100 | 4.90 | 9.39 | 4.39 | 8.96 |
|  | 625 | 7.79 | 6.37 | 7.46 | 6.62 |
|  | 1250 | 8.73 | 7.95 | 5.25 | 6.06 |

**Supplementary Table S4.** Extraction recovery and matrix effect results of 12 target substances

| **Substances** | **Theoretical concentration (ng/mL)** | **Extraction recovery** | | **matrix effects** | |
| --- | --- | --- | --- | --- | --- |
|  |  | **MEAN (%)** | **(CV%)** | **MEAN (%)** | **(CV%)** |
| L-Tyrosine | 50 | 87.7 | 4.80 | 87.2 | 4.06 |
|  | 200 | 86.6 | 4.18 | 88.0 | 4.81 |
|  | 500 | 72.0 | 1.62 | 98.9 | 2.83 |
| L-Phenylalanine | 50 | 82.9 | 3.63 | 95.0 | 6.95 |
|  | 200 | 106.0 | 7.56 | 91.3 | 12.50 |
|  | 500 | 75.2 | 3.78 | 87.0 | 7.82 |
| L-Valine | 50 | 66.7 | 3.55 | 109.0 | 4.21 |
|  | 200 | 63.4 | 3.55 | 89.3 | 2.04 |
|  | 500 | 64.8 | 4.92 | 95.3 | 4.47 |
| L-Lactic acid | 50 | 63.6 | 3.39 | 91.6 | 5.79 |
|  | 200 | 66.1 | 6.74 | 93.6 | 5.65 |
|  | 500 | 66.5 | 7.56 | 101.0 | 7.79 |
| Arachidonic acid | 50 | 76.4 | 5.12 | 89.3 | 4.06 |
|  | 250 | 77.0 | 10.20 | 92.6 | 4.06 |
|  | 500 | 78.2 | 5.44 | 94.3 | 5.91 |
| Linoleic acid | 100 | 70.3 | 3.54 | 93.9 | 7.98 |
|  | 500 | 83.2 | 5.63 | 99.0 | 5.69 |
|  | 1000 | 82.1 | 5.10 | 103.0 | 1.98 |
| Oleic acid | 100 | 79.8 | 7.91 | 92.2 | 5.38 |
|  | 500 | 85.9 | 6.66 | 101.0 | 8.10 |
|  | 1000 | 81.2 | 11.70 | 107.0 | 4.24 |
| Stearic acid | 100 | 80.5 | 11.60 | 98.9 | 7.98 |
|  | 625 | 90.3 | 9.06 | 97.7 | 4.67 |
|  | 1250 | 84.2 | 6.08 | 93.3 | 3.74 |
| Palmitic acid | 100 | 87.1 | 8.49 | 99.5 | 9.58 |
|  | 625 | 88.4 | 6.60 | 103.0 | 4.36 |
|  | 1250 | 92.1 | 10.30 | 103.0 | 4.52 |

**Supplemental Table S5.** Repeated freeze-thaw stability and short-term stability results of 12 target substances

| **Substance** | **Theoretical concentration (ng/mL)** | **Repeated freeze-thaw stability** | | | **short-term stability** | | |  |
| --- | --- | --- | --- | --- | --- | --- | --- | --- |
|  |  | **MEAN**  **(ng/mL)** | **(CV%)** | **RE (%)** | **MEAN**  **(ng/mL)** | **(CV%)** | **RE (%)** |  |
| L-Tyrosine | 50 | 55.2 | 2.43 | 7.46~13.9 | 54.4 | 3.03 | 6.07~12.6 |  |
|  | 200 | 202.0 | 3.68 | 0.064~5.73 | 202.0 | 3.22 | 0.355~4.51 |  |
|  | 500 | 517.0 | 2.76 | 1.35~6.25 | 522.0 | 2.79 | 0.570~6.59 |  |
| L-Phenylalanine | 50 | 44.8 | 2.98 | 6.65~13.0 | 44.4 | 2.82 | 8.12~14.4 |  |
|  | 200 | 214.0 | 5.80 | 2.19~14.5 | 218.0 | 3.86 | 2.06~13.2 |  |
|  | 500 | 489.0 | 5.47 | 1.16~7.23 | 473.0 | 4.79 | 2.33~8.69 |  |
| L-Valine | 50.0 | 48.8 | 4.71 | 2.09~5.68 | 47.0 | 2.00 | 3.18~7.51 |  |
|  | 200 | 184.0 | 1.16 | 6.97~9.57 | 180.0 | 2.44 | 7.01~11.9 |  |
|  | 500 | 529.0 | 2.80 | 2.87~10.7 | 540.0 | 2.43 | 5.76~11.7 |  |
| L-Lactic acid | 50 | 52.8 | 3.05 | 2.13~9.06 | 53.4 | 2.97 | 4.16~10.4 |  |
|  | 200 | 181.0 | 4.28 | 4.13~14.0 | 182.0 | 5.21 | 2.17~14.0 |  |
|  | 500 | 492.0 | 4.38 | 1.24~6.40 | 517.0 | 2.96 | 1.51~5.75 |  |
| Arachidonic acid | | 50 | 45.3 | 4.95 | 6.75~14.9 | 44.9 | 4.49 | 4.42~14.0 |
|  |  | 250 | 272.0 | 4.65 | 1.16~13.7 | 242.0 | 6.08 | 1.73~12.3 |
|  |  | 500 | 560.0 | 2.86 | 8.08~14.6 | 554.0 | 1.92 | 8.32~13.5 |
| Linoleic acid | | 100 | 91.2 | 4.88 | 3.98~14.3 | 90.1 | 2.13 | 7.10~11.4 |
|  |  | 500 | 446.0 | 2.38 | 7.19~12.4 | 449.0 | 3.33 | 6.56~14.1 |
|  |  | 1000 | 1125 | 1.28 | 10.2~14.1 | 1112.0 | 1.38 | 9.57~13.1 |
| Oleic acid | | 100 | 97.1 | 10.0 | 0.487~13.2 | 92.7 | 6.87 | 1.52~12.3 |
|  |  | 500 | 447.0 | 4.21 | 5.76~14.1 | 438.0 | 2.32 | 9.62~14.8 |
|  |  | 1000 | 1095.0 | 3.12 | 6.01~13.5 | 1063.0 | 6.97 | 0.831~12.5 |
| Stearic acid | | 100 | 109.0 | 4.13 | 2.13~12.9 | 108.0 | 7.33 | 4.13~13.9 |
|  |  | 625 | 618.0 | 7.40 | 0.59~8.88 | 596.0 | 9.65 | 5.83~11.9 |
|  |  | 1250 | 1381.0 | 2.75 | 5.45~12.8 | 1302.0 | 5.11 | 2.23~12.5 |
| Palmitic acid | | 100 | 111.0 | 1.76 | 9.02~13.3 | 104.0 | 6.97 | 3.20~8.84 |
|  |  | 625 | 582.0 | 11.4 | 9.65~13.9 | 599.0 | 6.94 | 1.34~10.1 |
|  |  | 1250 | 1262.0 | 7.98 | 2.02~14.6 | 1231.0 | 8.22 | 2.57~10.6 |
| LysoPC(18:0) | | 100 | 111.0 | 2.48 | 8.36~14.0 | 108.0 | 4.16 | 2.76~13.3 |
|  |  | 500 | 451.0 | 3.69 | 4.92~13.7 | 446.0 | 3.34 | 6.22~13.3 |
|  |  | 1000 | 955.0 | 5.84 | 0.784~11.0 | 1031.0 | 5.80 | 1.04~12.5 |
| LysoPC(18:1(9Z)) | | 100 | 89.5 | 4.23 | 5.47~14.1 | 89.8 | 3.50 | 6.60~14.2 |
|  |  | 500 | 442.0 | 2.98 | 7.87~14.7 | 457.0 | 9.93 | 6.46~14.8 |
|  |  | 1000 | 1002.0 | 8.68 | 3.77~13.8 | 1033.0 | 5.92 | 1.30~8.92 |
| LysoPC(16:0) | | 100 | 111.0 | 2.54 | 6.26~13.5 | 112.0 | 2.19 | 8.19~14.2 |
|  |  | 625 | 620.0 | 7.03 | 1.81~8.22 | 574.0 | 6.58 | 1.57~15.0 |
|  |  | 1250 | 1290.0 | 7.63 | 1.58~14.5 | 1380.0 | 2.38 | 7.72~13.9 |
